# Low-dose interleukin-2 induces clonal expansion of BACH2-repressed effector regulatory T cells following acute coronary syndrome

**DOI:** 10.1038/s44161-025-00652-y

**Published:** 2025-06-03

**Authors:** A. G. Case, J. W. O’Brien, Y. Lu, F. T. W. Charlier, X. Zhao, Y. Weng, L. Masters, Z. K. Tuong, R. Sriranjan, J. Cheriyan, C. Kemper, M. R. Clatworthy, Z. Mallat, T. X. Zhao

**Affiliations:** 1 Department of Medicine, University of Cambridge, Cambridge, UK; 2 NHLBI, National Institutes of Health, Bethesda, MD USA; 3 Cardiovascular Trials Office, Cambridge Clinical Trials Unit, Cambridge University Hospitals NHS Foundation Trust, Cambridge, UK; 4 Cambridge Institute for Therapeutic Immunology and Infectious Diseases, Cambridge, UK; 5 Université Paris Cité, Institut National de la Santé et de la Recherche Médicale, U970, PARCC, Paris, France; 6 Department of Cardiology, Royal Papworth NHS Foundation Trust, Cambridge, UK

**Keywords:** Myocardial infarction, Lymphocyte activation, Translational research

## Abstract

Targeting inflammation in atherosclerotic cardiovascular disease remains a major unmet need. Low-dose interleukin-2 (IL-2_LD_) selectively increases regulatory T (T_reg_) cell numbers in patients with coronary artery disease. Here we combine single-cell transcriptomics and T cell receptor analyses and show that IL-2_LD_ clonally expands effector T_reg_ cells in patients with acute coronary syndromes. The clonally expanded T_reg_ cells upregulate key immunosuppressive and metabolic pathways and show an increased number of predicted ligand–receptor interactions. These T_reg_ cells also display similar predicted antigen specificities, which cluster with published sequences specific to atherosclerotic cardiovascular disease. By tracking the T cell receptors of single cells over time, we identify an inflammatory polarization of the T cell compartment after myocardial infarction, which is restrained by IL-2_LD_. We identify BACH2 as a repressor of the T_reg_ effector program. However, BACH2-mediated regulation is bypassed with IL-2_LD_. Overall, these results lend insight into the IL-2-driven clonal expansion program in human T_reg_ cells, with important therapeutic implications for patients with cardiovascular and other immune-mediated diseases.

## Abstract (web summary)

By integrating single-cell transcriptomics and T cell receptor repertoire analyses of regulatory T (T_reg_) cells in a cohort of patients with acute coronary syndrome from the LILACS trial, Case et al. show that low-dose interleukin-2 clonally expands T_reg_ cells and maintains their suppressive program by bypassing BACH2 downregulation.

## Main

Chronic inflammation is a hallmark of atherosclerosis, with plaque formation resulting from excessive immune cell recruitment and activation following damage to the arterial wall^1^. The atherosclerotic plaques formed can rupture or erode, causing myocardial infarction (MI) and stroke^2^. The immune response triggered by the tissue injury further accelerates atherosclerosis progression and precludes cardiac repair, contributing to ischemic heart failure^3^. Despite the consequences of this unresolved inflammation for patients, colchicine is the only Food and Drug Administration-approved anti-inflammatory treatment for high-risk patients with atherosclerotic cardiovascular disease. However, recent negative data from the CLEAR trial, which showed no benefit of colchicine following acute MI, has cast doubts on colchicine’s efficacy^4^.

The role of adaptive immunity, particularly T cells, in atherosclerotic cardiovascular disease has attracted increased attention. Specifically, regulatory T (T_reg_) cells, which promote self-tolerance and regulate effector cell activation, have been investigated as a potential therapeutic target^5^. T_reg_ cells are atheroprotective and improve cardiac remodeling after MI in mice, while low T_reg_ cell levels are associated with an increased risk of MI in patients^5,6^. Interleukin-2 (IL-2) is essential for T_reg_ cell proliferation, maintenance and function^7,8^. While effector T (T_eff_) cells and natural killer cells express the dimeric, intermediate-affinity IL-2 receptor (IL-2R), T_reg_ cells express the high-affinity trimeric IL-2R containing IL-2Rα (CD25), resulting in low doses of IL-2 preferentially activating T_reg_ cells^8^.

The detailed impact of IL-2 treatment on T_reg_ cell function has been evaluated primarily using mouse studies^8,9^. Therefore, beyond the impact of IL-2_LD_ on T_reg_ cell levels and its potential association with clinical outcomes, new translational studies will be instrumental in elucidating the molecular mechanisms and phenotype switching associated with T_reg_ cell modulation by IL-2_LD_ in patients^10,11^. Furthermore, the effect of IL-2 on the T cell receptor (TCR) landscape in humans remains largely unexplored.

The Low-Dose Interleukin-2 in Patients with Stable Ischemic Heart Disease and Acute Coronary Syndromes (LILACS) study was a first-in-class, two-part, randomized, double-blind, placebo-controlled clinical trial that showed the safety and efficacy of IL-2_LD_ in patients with stable ischemic heart disease (Part A) and acute coronary syndrome (ACS) (Part B)^10^. In this previously published clinical trial, we focused on the effect of IL-2_LD_ on T_reg_ cell numbers and clinical safety. In this study, we evaluate the phenotype and immune receptors of T cells that were clonally expanded after IL-2_LD_ treatment following MI. By using paired single-cell T cell receptor sequencing (scTCR-seq) and single-cell RNA sequencing (scRNA-seq) data, we provide deeper insight into the impact of IL-2_LD_ therapy on T_reg_ cell maintenance and function and its effect on the TCR repertoire in patients with ACS.

## Results

### TCR landscape in the LILACS trial

**Fig. 1:**
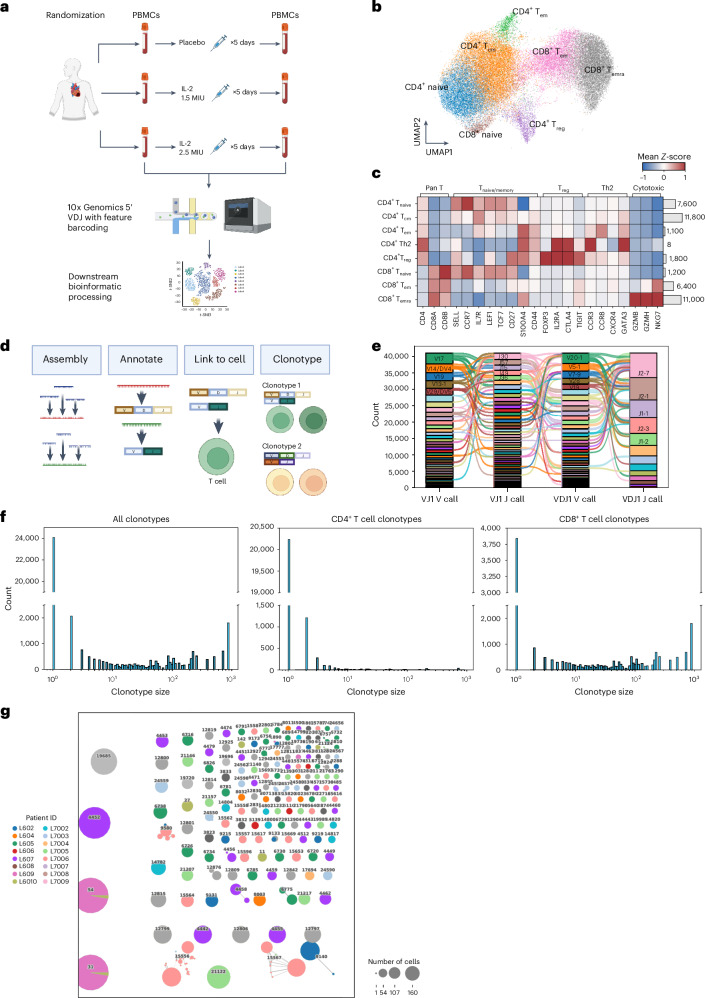
Unsupervised clustering of T cells from scRNA-seq data shows distinct T cell subsets and diverse clonotypes. **a**, LILACS study sample collection and workflow schematic. Three treatment groups from Part B of LILACS are evaluated in this study: placebo (*n* = 4), 1.5 MIU d^−1^ IL-2 (*n* = 6) and 2.5 MIU d^−1^ IL-2 (*n* = 6). **b**, Uniform manifold approximation and projection (UMAP) visualization of unsupervised clustering revealed 8 distinct T cell populations (*n* = 41,050 cells). **c**, Heat map with the average expression of canonical T cell function-associated genes. The histogram on the right of the heat map shows the number of cells within each cluster. **d**, Single-cell TCR workflow schematic. **e**, Gene segment usage and gene–gene pairing landscapes are illustrated graphically using four vertical stacks (one for each V and J segment) connected by curved segments with thickness proportional to the number of TCRs with the respective gene pairing. Each stack has genes with the highest frequencies stacked on top with subsequent genes in descending order. **f**, Plot showing the frequency of clonotype sizes across all T cells, CD4^+^ T cells or CD8^+^ T cells. **g**, Network analysis showing larger (*n* ≥ 15) clonotypes. Each number is a clonotype identification number, each individual color represents a single patient and the size of the dots represents the number of cells. Linked dots are clonotypes that are closely related. Panels **a** and **d** created with BioRender.com.

Peripheral blood mononuclear cells (PBMCs) were sampled from patients in the LILACS trial presenting with ACS before and after treatment with either placebo or IL-2_LD_. These PBMCs were processed to generate scTCR and scRNA-seq data and then analyzed to elucidate the impact of IL-2_LD_ on T cells and the TCR landscape (Fig. 1a). We re-clustered T cells into eight distinct populations based on canonical markers (Fig. 1b,c). Next, the paired scTCR-seq and scRNA-seq data were processed according to the workflow outlined in Fig. 1d. From the entire sequenced scTCR dataset (92,941 cells), there were 55,609 cells (60%) with single paired productive αβ chains, and 557 cells with multiple TCRα chains and/or β chains (0.60%). After exclusion of non-αβ T cells and merging with the scRNA-seq gene expression data, 41,050 single cells with single paired productive αβ chains were identified and brought forward for analysis. The resulting cells showed diverse V and J gene usage among the TCR repertoire (Fig. 1e). For a broad overview of the clonotype landscape, the frequency of clonotype sizes was plotted, where a clonotype is defined by cells that share a highly similar TCR CDR3 sequence (Methods and Fig. 1f). The majority of clonotypes were unique, that is, they had a clonotype size of 1, while clonotype sizes ranged from 1 to 909. These results also confirm previous findings that the clonotype landscape in human T cells is dominated by CD8^+^ rather than CD4^+^ T cells, and that CD8^+^ terminally differentiated effector memory (T_emra_) cells comprise the largest clonotypes^12^. The highly expanded clonotypes, defined as those with 15 or more component cells (number of cells = 11,305), were then examined using a network analysis in which node colors correspond to individual patients. The network analysis shows that the majority of clonotypes are private, that is, restricted to individual patients (Fig. 1g). As we have previously shown that IL-2_LD_ selectively expanded and activated CD4^+^ T_reg_ cells, which is suggested to be the most likely immunomodulatory mechanism of IL-2_LD_, our subsequent analysis focuses primarily on the CD4^+^ T cell compartment.

### The effect of IL-2_LD_ on the TCR landscape

**Fig. 2:**
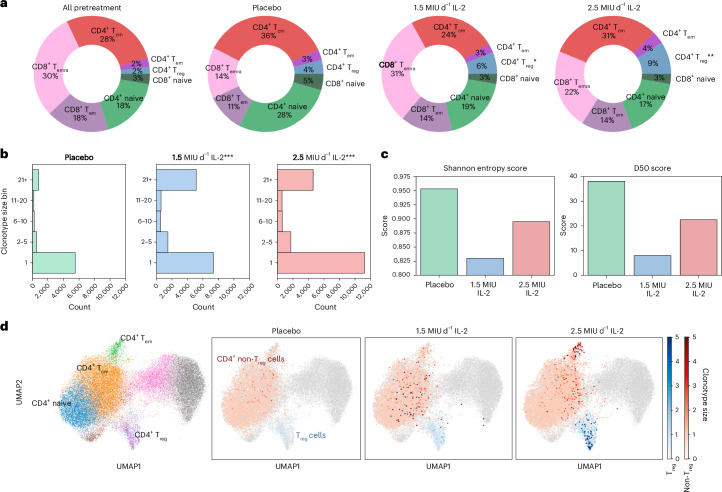
The effect of IL-2_LD_ on the CD4^+^ T cell proportions and TCR landscape. **a**, scRNA-seq data showing the effect of IL-2 versus placebo on T cell subsets. There was a significant increase in T_reg_ cell numbers after 1.5 MIU d^−1^ and 2.5 MIU d^−1^ of IL-2 treatment compared with pooled pretreatment counts: *P = 0.034; **P = 0.0072 by two-tailed *t*-test. **b**, Histogram showing the distribution of clonotype size across 5 bins: 1, 2–5, 6–10, 11–20 and 21+. The bars represent the proportion of that clonotype size in each treatment allocation. ***P < 0.001 (by chi-squared (one-tailed) test), indicating significant difference in clonotype size proportions between 1.5 MIU d^−^^1^ or 2.5 MIU d^−1^ doses and placebo. **c**, Histograms showing the effect of the treatment allocations on TCR diversity using Shannon entropy and D50. **d**, The left panel shows a UMAP with the CD4^+^ T cell subsets overlaid. The other panels show the effect of either placebo or IL-2 treatment on CD4^+^ T cell clonotype size. The shades of red or blue represent the size of clonotypes on a scale from 0 to >5. Clonotype size is defined as the number of CD4^+^ T cells that share highly similar TCR sequences (Methods).

The scRNA-seq T cell analysis showed that IL-2_LD_ significantly increased the percentage of T_reg_ cells in a dose-dependent manner (pretreatment, 2%; placebo, 4% (*P* = 0.927, *t* = 0.0923, d.f. = 38); 1.5 MIU d^−1^, 6% (*P* = 0.0336, *t* = 2.197, d.f. = 42); 2.5 MIU d^−1^, 9% (*P* = 0.0072, *t* = 2.824, d.f. = 42); two-tailed *t*-tests) (Fig. 2a). In our previous analysis, we assessed clonal expansion using the entire TCR repertoire^10^; here we evaluate only those TCRs associated with CD4^+^ T cells. When assessing the clonotype size of all CD4^+^ T cells across the placebo and treatment groups, it was observed that IL-2_LD_ causes a significant decrease in the proportion of unique clonotypes (size = 1) with a corresponding increase in large clonotypes in both IL-2_LD_ treatment groups (*P* < 0.001, *x*² = 4,294.07, d.f. = 59; *P* < 0.001, *x*² = 3,429.84, d.f. = 69; multiple chi-squared tests for the 1.5 MIU d^−1^ and 2.5 MIU d^−1^ groups, respectively) (Fig. 2b). Correspondingly, IL-2_LD_ treatment caused a decrease in TCR diversity as measured by Shannon entropy and diversity index (D50; Fig. 2c). Furthermore, patients with acute MI treated with placebo had a limited expansion of CD4^+^ T cell clonotypes, while patients treated with 1.5 MIU d^−1^ of IL-2 had an expansion of T_reg_ cells as well as naive and central memory T (T_cm_) cell clonotypes. Patients treated with 2.5 MIU d^−1^ of IL-2 had a marked expansion of both T_reg_ cells and effector memory T (T_em_) cell clonotypes (Fig. 2d).

### TCR tracking

**Fig. 3:**
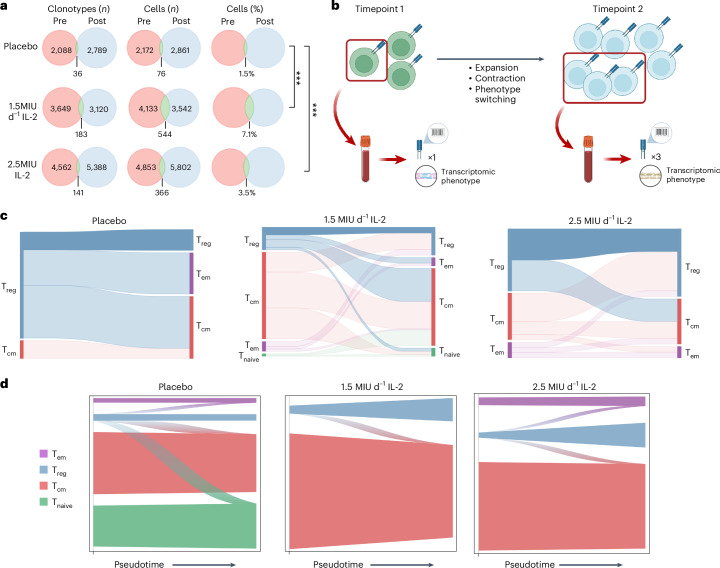
The effect of IL-2_LD_ on scTCR matching between the pre- and post-dose timepoints. **a**, Venn diagrams showing the overlap of CD4^+^ TCR clonotypes (and associated T cells) that match between the pre- and post-dose timepoints in the various treatment allocations. ***P < 0.001 (by chi-squared (one-tailed) test), indicating a significant difference. **b**, Schematic showing the concept of TCR tracking. We use the TCR as a barcode to track cells over two timepoints before and after dosing. By then pairing scTCR-seq and scRNA-seq, we can phenotype these tracked cells. **c**, Using TCR tracking, we show the effect of acute MI (treated with placebo) on matched TCRs and their cell phenotype over two timepoints. Connected lines from the pre- and post-dose timepoints represent CD4^+^ T cells that have the same TCR clonotype. The labels on the sides describe the scRNA-seq phenotype of the cell. Only those clonotypes tracked onto T_reg_ cells at either the pre- or post-dose timepoints are shown. The numbers of cells underlying this figure are provided in Supplementary Table 1. **d**, Results of transition matrix calculation within each treatment group followed by outgoing flow visualization using CellRank 2. Panel **b** created with BioRender.com.

**Extended Data Fig. 1:**
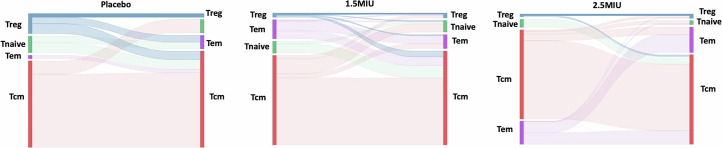
Scaled TCR Tracking Sankey Plots. Sankey plots showing cell tracking over time via TCR normalized to absolute cell counts (see Supplementary Table 1). The left column of each diagram represents the pre-treatment timepoint, and the right column the post-treatment timepoint. The connecting lines in between denote TCR tracking.

**Extended Data Fig. 2:**
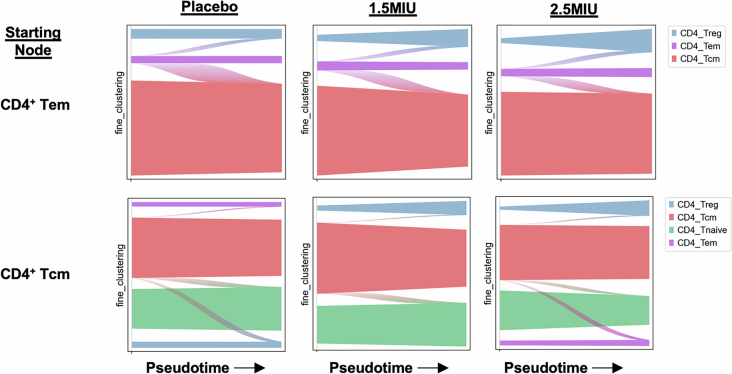
Additional CellRank 2 Analysis. Results of transition matrix calculation within each treatment group followed by outgoing flow visualization using CellRank 2. Starting node cell types are Tem = effector memory T cells or Tcm = central memory T cells.

Focusing on TCRs before (pre) and after (post) treatment with placebo or IL-2_LD_, we analyzed the number of shared clonotypes detected between the two timepoints. The numbers of shared clonotypes in the placebo, and 1.5 MIU d^−1^and 2.5 MIU d^−1^ groups, were 36, 183 and 141, respectively. With regard to cells with shared clonotypes, there was a significantly higher proportion in the IL-2_LD_-treated groups than in the placebo-treated groups (placebo = 76 (1.5%), 1.5 MIU d^−1^ = 544 (7.1%) and 2.5 MIU d^−1^ = 366 (3.5%); *P* < 0.001, *x*² = 327.8, d.f. = 2; *P* < 0.001, *x*² = 51.99, d.f. = 2, respectively; multiple chi-squared tests) (Fig. 3a). We leverage the fact that there are more possible TCR combinations compared with the number of circulating T cells in the body^13^, and use the TCR as a unique barcode. Tracking this barcode across the two timepoints facilitates inference about whether a particular clonotype associated with a given set of T cells has expanded or contracted. Furthermore, by linking the TCR to the individual single-cell transcriptome, specific changes in T cell phenotypes can be assessed (Fig. 3b). In the natural progression of acute MI (patients treated with placebo), and considering only those clonotypes tracked onto T_reg_ cells at either the before or after timepoints, we see an overall statistically significant contraction of the T_reg_ cell compartment paired with expansion of the T_em_ cell compartment: 6 T_reg_ cells and 1 T_cm_ cell at timepoint 1 tracked 1 week later to 1 T_reg_, 3 T_cm_ and 2 T_em_ cells at timepoint 2 (*P* = 0.0414, Fisher’s exact test; Fig. 3c and Supplementary Table 1). This shift from a regulatory to an effector phenotype is consistent with the inflammatory milieu observed in post-acute MI patients^14^. In patients treated with IL-2_LD_, retention of the regulatory milieu is observed. For example, in the 2.5 MIU d^−1^ treatment group, 4 T_reg_ cells, 3 T_cm_ cells and 1 T_em_ cell are tracked to 6 T_reg_ cells, 4 T_cm_ cells and 1 T_em_ cell, indicating that IL-2_LD_ maintains the T_reg_ cell phenotype in the post-MI setting. TCR tracking counts for all clonotypes shared between timepoints in each treatment condition are summarized in Extended Data Fig. 1 and Supplementary Table 2 with the full database of expanded CDR3s contained in Supplementary Table 3. To corroborate these TCR tracking results across the wider dataset, a transition matrix was calculated within each treatment group, and the outgoing flow from each cluster of cells was visualized using CellRank 2 (ref. ^15^). This analysis also showed a polarization away from the T_reg_ cell phenotype in the placebo condition in favor of effector, central memory and naive T cells. In the two IL-2_LD_ treatment conditions, greater maintenance of (that is, reduced flow from) the T_reg_ cell cluster is confirmed (Fig. 3d). In addition, when the starting cell type is set to either T_cm_ or T_em_ cell, a relative polarization toward T_reg_ cells is seen with IL-2_LD_ treatment (Extended Data Fig. 2).

### TCR clustering and antigen specificity

**Fig. 4:**
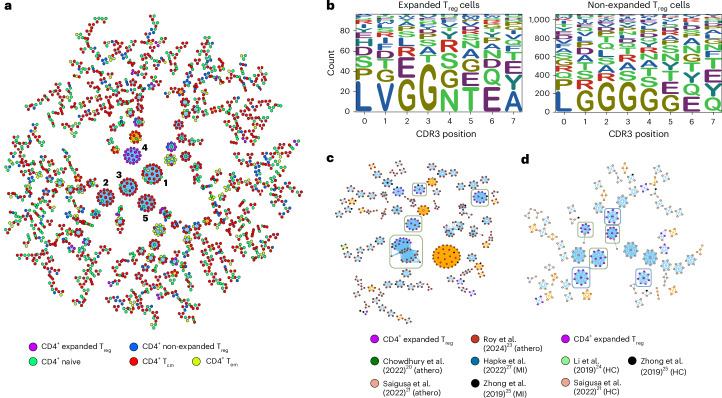
TCR motifs of clonally expanded T_reg_ cells cluster together. **a**, GLIPH2-based network analysis showing clusters based on TCR motifs. Each dot represents a TCR CDR3, the colors represent cell type and clustering is done by either local or global similarity depicted by yellow or blue lines. Global similarity (blue lines) allows differences at the same position between exchangeable amino acids according to a BLOSUM62 matrix. Local similarity (yellow lines) allows the position of the CDR3 motif to vary only within three amino acids. Larger clusters are mapped to the center while smaller clusters are mapped to the outside. The numbers are identifiers that link to the CDR3 motif found in Supplementary Table 4. **b**, WebLogo plots showing differences in aligned CDR3 composition in clonally expanded versus non-expanded T_reg_ cells. The height of the letters represents relative frequency, with decreasing frequency stacked on top. **c**, GLIPH2-based network analysis showing clusters based on TCR motifs with our expanded T_reg_ TCRs and other published papers^20,21,23,25,27^ (Supplementary Table 5) relating to atherosclerosis (labeled ‘athero’) and MI (labeled ‘MI’). **d**, Corresponding GLIPH2-based network analysis with healthy controls (HC)^21,24,25^. Only clusters with more than three CDR3 instances are plotted. The green boxes highlight examples of our expanded T_reg_ TCRs clustering with other datasets. The blue boxes highlight examples of our expanded T_reg_ TCRs clustering among themselves.

**Extended Data Fig. 3:**
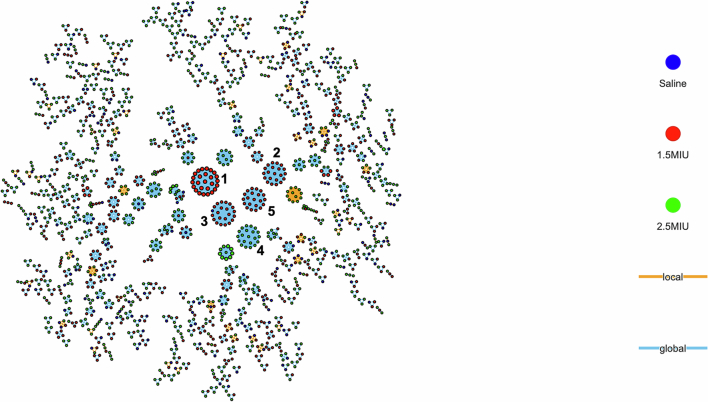
Additional GLIPH2 Analysis. GLIPH2-based network analysis showing clusters based on TCR motifs, coloured by treatment group (produced using TurboGLIPH). Labelled clusters correspond to data in Supplementary Table 4.

**Extended Data Fig. 4:**
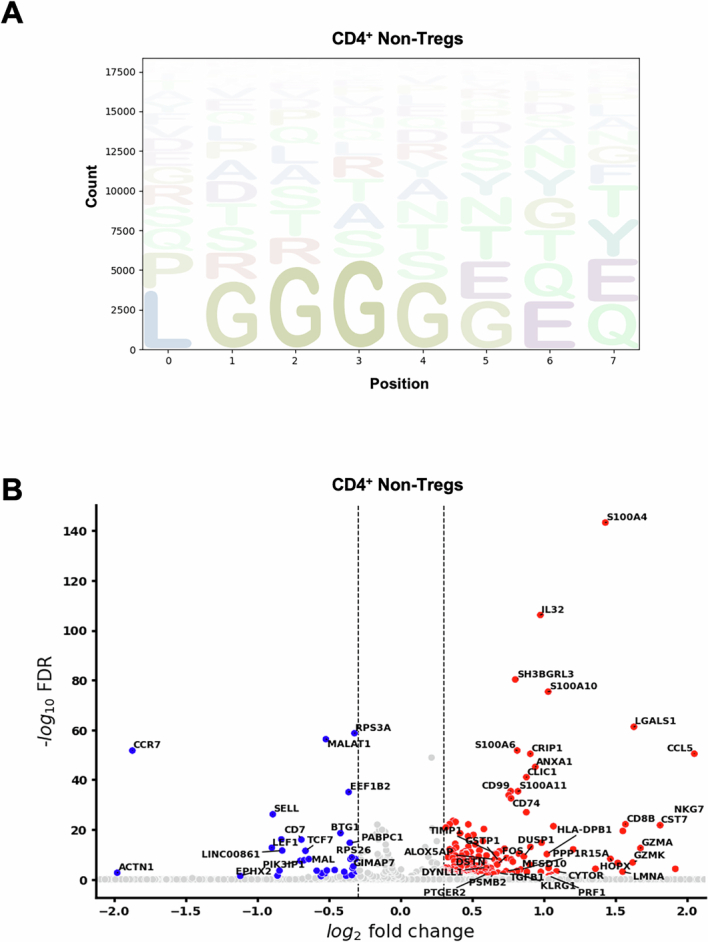
Non-Treg CD4^+^ T Cell Analysis. **A**, Weblogo plot showing differences in aligned CDR3 composition in clonally expanded CD4+ non-Tregs. The height of the letters represent the relative frequency with decreasing frequency stacked on top. **B**, Volcano plot showing differentially expressed genes in expanded CD4+ non-Tregs vs. nonexpanded CD4+ non-Tregs (enriched in the expanded (red) or non-expanded (blue).

**Extended Data Fig. 5:**
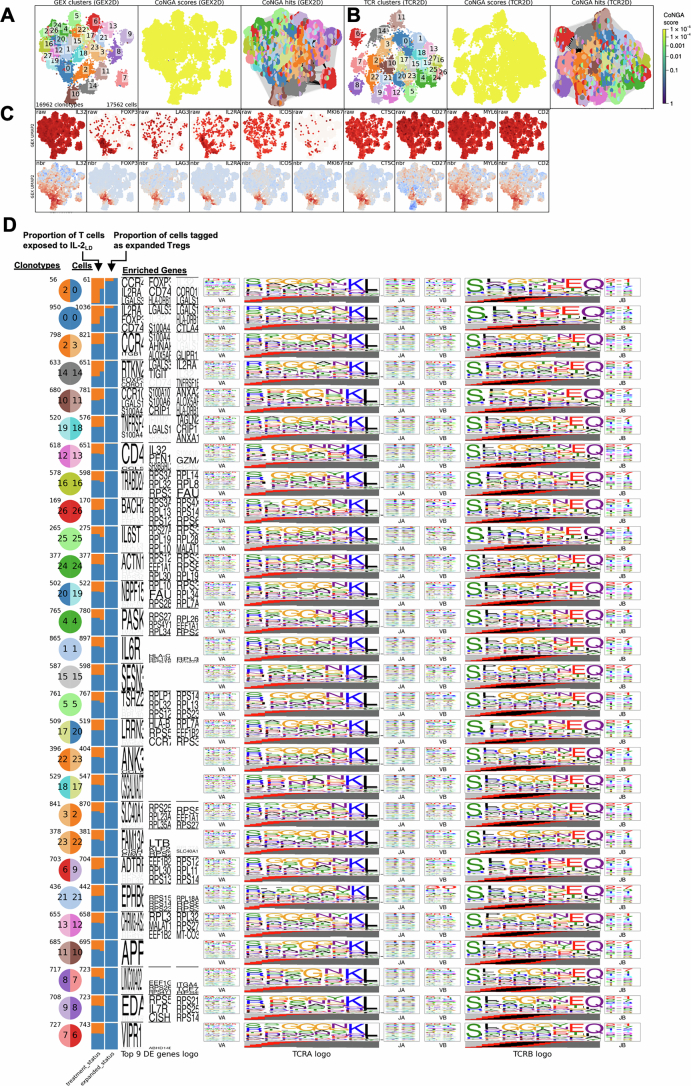
CoNGA Graph-vs-Graph Analysis. Clonotype Neighbor Graph Analysis (CoNGA) of CD4+ T cells. The Graph-vs-Graph Analysis module was used to produce panels **A**-**D**. At the top (**A** and **B**) are six 2D UMAP projections of clonotypes in the dataset based on GEX similarity (**A**) and TCR similarity (**B**), coloured from left to right by GEX cluster assignment; CoNGA score; joint GEX:TCR cluster assignment for clonotypes with significant CoNGA scores, using a bicolored disk whose left half indicates GEX cluster and whose right half indicates TCR cluster; TCR cluster; CoNGA; GEX:TCR cluster assignments for CoNGA hits, as in the third panel. Below (**C**) are two rows of GEX landscape plots coloured by (first row, left) expression of selected marker genes, (second row, left) Z-score normalized and GEX-neighborhood averaged expression of the same marker genes, and (both rows, right) TCR sequence features. In **D**, GEX and TCR sequence features of CoNGA hits are summarized by a series of logo-style visualizations, from left to right: differentially expressed genes (DEGs); TCR sequence logos showing the V and J gene usage and CDR3 sequences for the TCR alpha and beta chains; DEG and TCRseq sequence logos are scaled by the adjusted *p* value of the associations, with full logo height requiring a top adjusted *p* value below 10^−6^. DEGs with fold-change less than 2 are shown in grey. Each cluster is indicated by a bicolored disk coloured according to GEX cluster (left half) and TCR cluster (right half). The two numbers above each disk show the number of hits within the cluster (on the left) and the total number of cells in those clonotypes (on the right). The dendrogram at the left shows similarity relationships among the clusters based on connections in the GEX and TCR neighbor graphs. (Caption adapted from text in CoNGA Graph-vs-Graph Analysis output.)

Adaptive immunity is characterized by an antigen-specific response; therefore, we explored antigen specificity in the IL-2_LD_ clonally expanded T_reg_ cells. Several tools have been produced to cluster TCRs with shared specificities based on similar CDR3β motifs. The CDR3 region of the TCR has been shown to predominantly drive antigen specificity^16^. TurboGLIPH, which uses GLIPH2 (grouping of lymphocyte interactions by paratope hotspots), was used to produce a cluster analysis by plotting all CD4^+^ cells^17^. The analysis generated 11 clusters with clonotypes greater than 10, and all were from patients treated with IL-2_LD_. From these 11 clusters, 5 very large clusters with more than 20 TCRs emerged. Of the 5 clusters, 4 were from the 1.5 MIU d^−1^ group and 1 was from the 2.5 MIU d^−1^ group (Extended Data Fig. 3). Of the 5 large clusters, 4 were made of T_eff_ cells, which is expected given that they are the dominant CD4^+^ T cell population. However, unexpectedly, one of the very large clusters is composed of expanded T_reg_ cells (Fig. 4a; the corresponding list of annotated CDR3 motifs is provided in Supplementary Table 4). The CDR3 motif from the expanded T_reg_ cells was plotted using WebLogo and showed that, in aggregate, they display different sequence motifs compared with non-expanded T_reg_ cells (Fig. 4b). The CDR3 motif of clonally expanded CD4^+^ non-T_reg_ cells was also plotted, showing limited resemblance to that of the expanded T_reg_ cells (Extended Data Fig. 4a). To further investigate this finding, we used Clonotype Neighbor Graph Analysis (CoNGA), a recently developed graph theory-based workflow that identifies correlations between whole transcriptomic gene expression profiles and TCR sequences through statistical analysis of similarity graphs^18^. We show that IL-2_LD_-expanded T_reg_ cells cluster together in specific neighborhoods based on TCR similarity and are associated with an effector T_reg_ cell phenotype (Extended Data Fig. 5).

**Extended Data Fig. 6:**
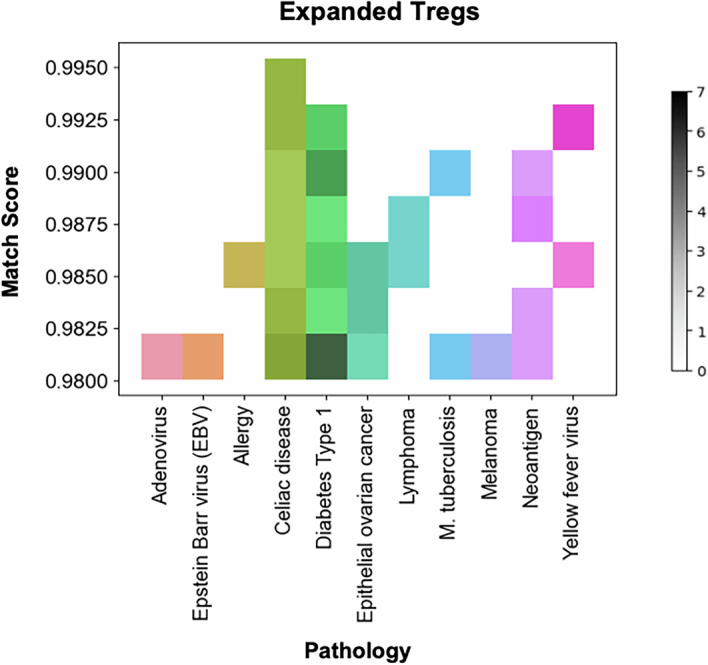
ERGO-II Antigen Prediction Results. Matrix showing the results of a large language model, ERGO-II, used to predict TCR-peptide binding of clonally expanded Tregs. Only match scores >0.980 have been plotted. The colour of the plot shows the pathological group the peptide sequence originated from. The intensity of the square colour represents the number of peptides used in the match.

Potential IL-2_LD_-expanded T_reg_ antigen identity was explored using two methods. First, we used ERGO-II, a sequence-based TCR–peptide binding predictor that uses deep learning-based methods incorporating both α and β chains, V and J genes, and the cell type to generate a binding score for a selected library of peptides^16^. The TCR sequences were extracted from the clonally expanded T_reg_ cells and searched against the McPAS database, which is a curated dataset of TCR–peptide binding pairs from various pathological conditions^19^. The output of the ERGO-II prediction is a list of possible interactions with a single peptide binding score that ranges from 0, indicating poor predicted binding, to 1, indicating perfect predicted binding. From this, only very high TCR–peptide binding scores (greater than 0.980) were evaluated (Extended Data Fig. 6). Several high match scores were observed for autoimmune diseases such as celiac disease and type 1 diabetes mellitus in addition to predicted recognition of general allergy and common viral antigens.

The McPAS database is primarily biased toward antigen specificities that are largely unrelated to cardiovascular disease. To analyze our data in the context of potential atherosclerotic cardiovascular disease-related antigens, we conducted a literature search and identified eight studies^20–27^ reporting TCR sequences associated with either atherosclerosis or MI (detailed in Supplementary Table 5). Importantly, four of the eight papers had in vitro validation of TCR–antigen specificity. We then used GLIPH2 to analyze TCR motif similarity by clustering TCRs from our expanded T_reg_ cells alongside those from the eight published papers. We visualize clusters that contain more than three CDR3s and show that the expanded T_reg_ cells cluster with TCRs previously confirmed via tetramer staining to be specific to ApoB (ref. ^23^). Notably, 93% of clustered expanded T_reg_ cells cluster with TCRs related to atherosclerotic cardiovascular diseases extracted from other sources (Fig. 4c). As a negative control, we performed the same analysis using TCRs from matched healthy controls, available from four of the eight studies (detailed in Supplementary Table 5). In this case, our expanded T_reg_ cells primarily formed clusters among themselves, separate from those of the healthy controls, with only 49% of clustered motifs clustering with TCRs from other sources (Fig. 4d).

### Phenotype of clonally expanded T cells

**Fig. 5:**
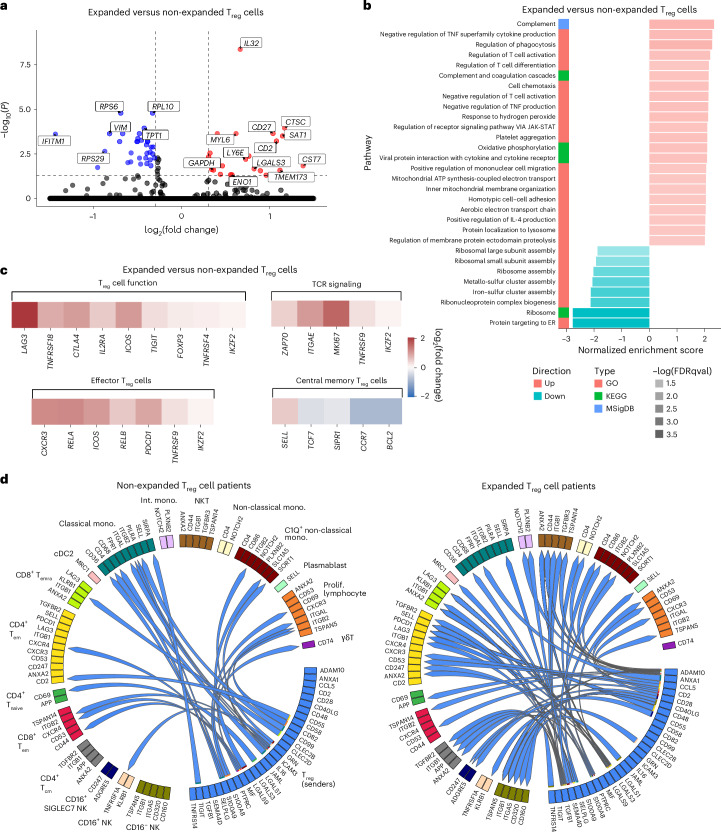
IL-2_LD_ clonally expanded T_reg_ cells are more activated and show an immunosuppressive phenotype. **a**, Volcano plot showing differentially expressed genes in expanded versus non-expanded T_reg_ cells (enriched in the expanded (red) or non-expanded T_reg_ cells (blue)). Genes within the vertical lines did not meet the effect size threshold (log_2_(fold change) > 0.3), and genes below the horizontal line did not meet the significance threshold (*P*_adj_  < 0.05). Statistical analysis was performed using a Wilcoxon rank-sum test (two sided) with Benjamini–Hochberg correction. **b**, GSEA assessing gene ontology pathways associated with biological processes in clonally expanded versus non-expanded T_reg_ cells. The red and blue bars represent upregulated and downregulated pathways, respectively, in the expanded T_reg_ cells. FDRqval, false discovery rate (FDR) q-value. **c**, Heat map showing the relative difference in expression of genes in the expanded T_reg_ cells compared with the non-expanded T_reg_ cells based on differential gene expression (expressed as log_2_(fold change)). **d**, Circos plot showing differences in prioritized interactions in patients with clonally expanded versus non-expanded T_reg_ cells as determined via MultiNicheNet analysis. Sender cells are set as T_reg_ cells, and possible receiving cells are set as other, non-T_reg_ immune cells. Classical mono., classical monocyte; Non-classical mono., non-classical monocyte; Int. mono., intermediate monocyte; C1Q^+^ non-classical mono., C1Q^+^ non-classical monocyte; cDC2, type-2 conventional dendritic cell; SIGLEC7 NK, Siglec-7 natural killer (NK) cell; γδT, γδ T cell; Prolif. lymphocyte, proliferating lymphocyte; NKT, NK T cell.

To more extensively phenotype these clonally expanded T_reg_ cells (clonotype size > 1, *n* = 127), differential gene expression and pathway analyses were used. The expanded T_reg_ cells were compared against non-expanded T_reg_ cells (clonotype size = 1, *n* = 1,103). From the differential gene expression results, many genes that were significantly upregulated (false discovery rate (FDR) < 0.05 and log_2_(fold change) > 0.3) were linked to T_reg_ activation and proliferation (Fig. 5a). These included *IL-32*, *CD27* and *LGALS3*. On the contrary, genes involved in restraining T_reg_ metabolism and suppressive activity, such as *VIM* (ref. ^28^), were significantly downregulated in expanded T_reg_ cells. With regard to cell metabolism, expanded T_reg_ cell upregulated genes related to both oxidative phosphorylation (*MT-CO1*, *MT-CO2*) and glycolysis (*GAPDH*, *ENO1*, *LGALS3*). The differential gene expression pattern of clonally expanded, CD4^+^ non-T_reg_ cells against non-expanded, CD4^+^ non-T_reg_ cells (Extended Data Fig. 4b) was also assessed. Generally, expanded non-T_reg_ cells did not show similar gene expression compared with expanded T_reg_ cells.

Pathway analysis using gene set enrichment analysis (GSEA) confirmed differential regulation of T_reg_ activation pathways as well as a substantial upregulation of complement and electron transport–oxidative phosphorylation pathways. (Fig. 5b). In addition, when evaluating gene signatures for different T_reg_ subsets and functions, clonally expanded T_reg_ cells showed greater expression of genes related to T_reg_ function (*LAG3*, *ICOS*, *IL-2RA*, *TNFRSF18*), proliferation and TCR activation (*ZAP70*, *MIK67*), as well as T_reg_ migration and tissue trafficking (*CXCR3*, *ITGAE*). Furthermore, they showed an effector as opposed to a central memory phenotype (Fig. 5c).

### Cell–cell interactions of expanded T_reg_ cells

**Extended Data Fig. 7:**
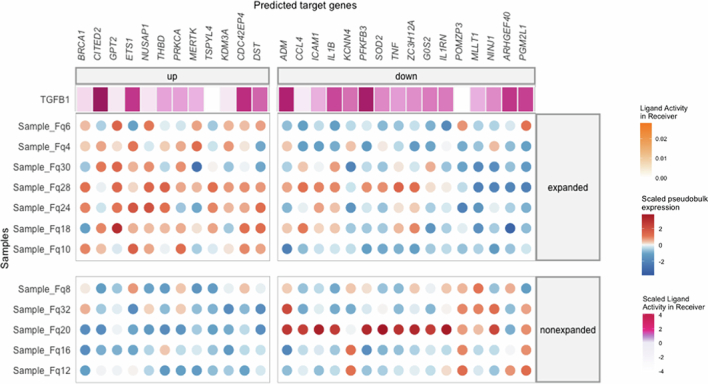
Extended MultiNicheNet Analysis Output. MultiNicheNet analysis comparing patients with expanded Tregs to those without. The sample-wise table shows post-timepoint samples from each patient and the predicted target gene pseudobulk expression values. These are grouped by patient expansion status.

Next, potential mechanisms by which expanded T_reg_ cells signal to other cell types were elucidated. For this, we used MultiNicheNet, a framework for differential cell–cell communication analysis that generates prioritization of ligand–receptor interactions through the evaluation of downstream gene activation of receptors in receiving cells^29,30^. Immune cell interactions were compared in an unbiased manner within two groups of patients, those with expanded T_reg_ clonotypes (sizes > 1) and those without expanded T_reg_ clonotypes (size = 1), at the posttreatment timepoint. We set T_reg_ cells as the sender cells (that is, ligand sources) and plotted the highest prioritized interactions (Fig. 5d). Patients with non-clonally expanded T_reg_ cells had fewer highly prioritized interactions overall. Contrastingly, patients with clonally expanded T_reg_ cells had more highly prioritized interactions onto multiple effector immune cell types via several well-known anti-inflammatory pathways. For example, transforming growth factor-β (TGF-β) signaling onto several cell subtypes including CD4^+^ and CD8^+^ T_eff_ cells is observed. Other notable ligand–receptor interactions upregulated in the expanded T_reg_ cell patients include ADAM10 to NOTCH2 and CD44 (refs. ^31,32^), CD2–CD53 (ref. ^33^), LGALS3–LAG3 (ref. ^34^) and CD48–PDCD1 (ref. ^35^). The MultiNicheNet analysis also outputs the downstream genes of receptor activation whose upregulation or downregulation supports the overarching ligand–receptor prediction (Extended Data Fig. 7).

### Regulation of T_reg_ cell expansion in response to IL-2_LD_ treatment

**Fig. 6:**
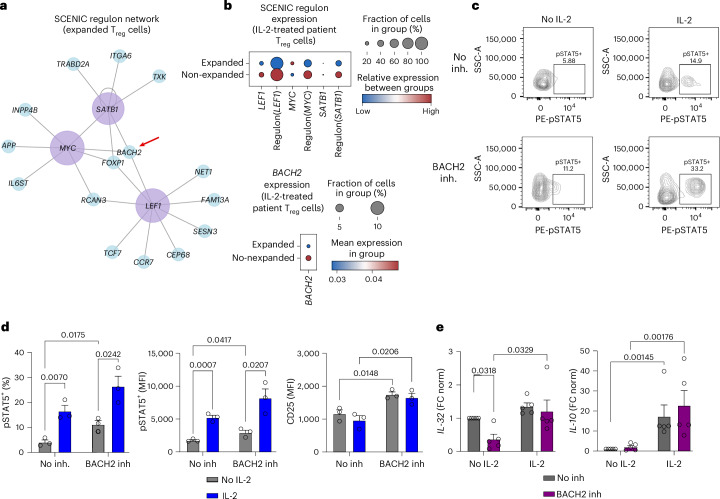
IL-2 treatment maintains the T_reg_ cell phenotype through a mechanism independent of BACH2 regulation. **a**, Network plot from regulon analysis of expanded versus non-expanded T_reg_ cells using SCENIC. **b**, Expression of *BACH2* and encompassing regulons in expanded versus non-expanded T_reg_ cells from the LILACS dataset. **c**, Representative data from flow cytometry analysis (*n* = 3) of pSTAT5 levels in primary human T_reg_ cells after 48 h of stimulation and an 8-h rest in serum-free media ± BACH2 inhibitor (inh.) (RGFP966), followed by 15 min of IL-2 treatment. Numeric values indicate percentages. SSC, side scatter; MFI, mean fluorescence intensity; FC norm, normalized fold change. **d**, pSTAT5 and CD25 levels assessed via flow cytometry (*n* = 3). **e**, RT-qPCR analysis of *IL-32* (*n* = 5) and *IL-10* (*n* = 5) after 24 h in primary human T_reg_ cells treated with BACH2 inhibitor and/or IL-2 (error bars represent s.e.m.; *P* values were determined by two-tailed independent *t*-test; *n* = number of independent experiments). Source data

**Extended Data Fig. 8:**
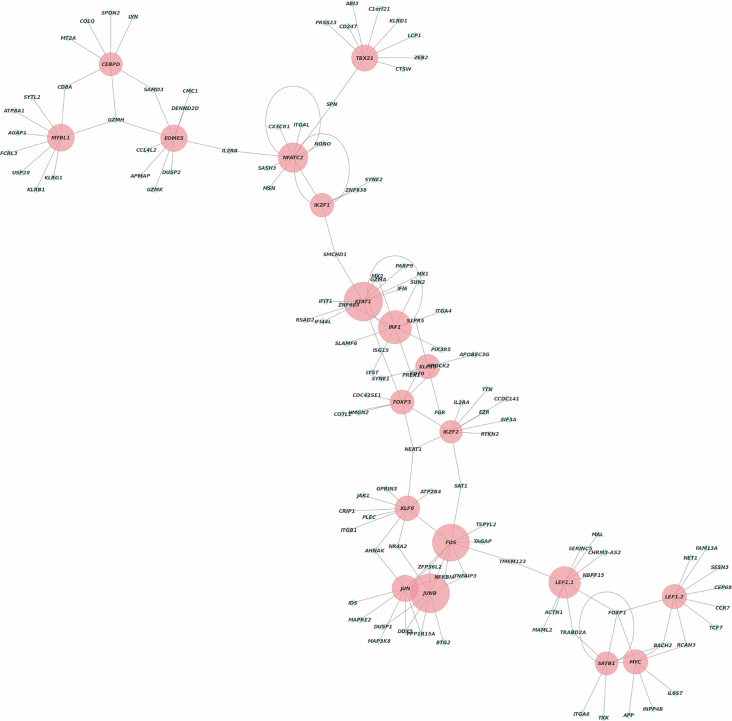
SCENIC Network Analysis. Network plot showing statistically significant (*p* < 0.05) regulons resulting from SCENIC analysis of expanded Tregs compared to non-expanded Tregs. Network generated using the derive_regulons() function. Regulon names are noted in red, and component genes are noted in blue.

Next, we investigated the transcriptional regulators of T_reg_ cell expansion in response to IL-2_LD_ treatment using single-cell regulatory network inference and clustering (SCENIC)^36^. A comparison between non-expanded and clonally expanded IL-2-treated T_reg_ cells yielded a network of several differentially expressed regulons (Extended Data Fig. 8), with MYC, SATB1 and LEF1 being further analyzed owing to shared regulon components (Fig. 6a). *BACH2* was identified as a common gene in these regulons and was downregulated along with lead regulon genes (that is, *SATB1* and *LEF1*) in the clonally expanded T_reg_ cells from IL-2_LD_-treated patients (Fig. 6b). These findings are highly relevant given the important role of BACH2 in T_reg_ cell differentiation and function. In mice, while BACH2 suppression is associated with an effector T_reg_ cell phenotype^37,38^, BACH2 plays an essential role in T_reg_ cell formation^39^, and deletion of BACH2 in T_reg_ cells is associated with the development of autoimmunity^40^. However, the role of BACH2 in human T_reg_ cells is poorly understood. The downregulation of BACH2 in IL-2_LD_-expanded T_reg_ cells is highly consistent with the enrichment of the effector T_reg_ cell program in these cells. However, long-term downregulation of BACH2 in T_reg_ cells of patients with coronary artery disease may be harmful given the essential role of BACH2 in T_reg_ cell formation. We therefore assessed the impact of in vitro BACH2 inhibition on the phenotype of human T_reg_ cells, and its modulation by IL-2.

**Extended Data Fig. 9:**
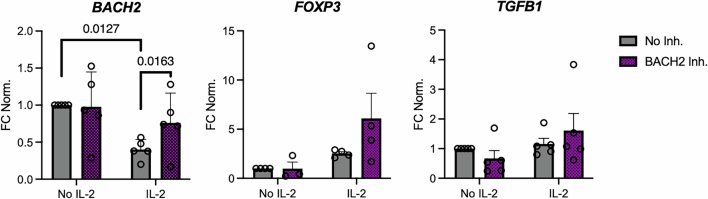
Primary Human Treg RT-qPCR Results. RT-qPCR analysis of *BACH2* (n = 5), *FOXP3* (n = 3 (No IL-2), n = 4 (IL-2)), and *TGFB1* (n = 5) after 24 h of cell culture with IL-2 and/or BACH2 inhibitor-treatment in primary anti-CD3/anti-CD28 stimulated human Tregs (error bar represents SEM, n = number of independent experiments, *p*-values determined by two-tailed independent t-test). Source data

To evaluate whether IL-2 treatment directly affects *BACH2* expression, isolated primary human T_reg_ cells were stimulated with anti-CD3 and anti-CD28 antibodies and cultured for 24 h with IL-2. Quantitative reverse transcription polymerase chain reaction (RT-qPCR) analysis confirmed significant downregulation of *BACH2* in response to IL-2 (*P* = 0.0127, *t* = 3.194, d.f. = 8; two-tailed independent *t*-test), in agreement with the in vivo data and supporting a direct effect (Extended Data Fig. 9). We then assessed the impact of BACH2 inhibition on STAT5 phosphorylation and CD25 surface expression in anti-CD3- and anti-CD28-stimulated primary human T_reg_ cells, in the presence or absence of IL-2. Expression levels of pSTAT5 were greater in cells treated with the BACH2 inhibitor (RGFP966) compared with those in non-inhibited cells (Fig. 6c), and this was most probably because of increased CD25 expression levels in the BACH2-inhibited conditions (Fig. 6d). With IL-2 treatment, the difference in pSTAT5 levels between the inhibited and non-inhibited conditions was maintained (Fig. 6c,d). We then examined whether BACH2 inhibition may affect the response of T_reg_ cells to IL-2. In patients with MI treated with IL-2_LD_, *IL-32* was one of the prominent differentially expressed genes in the expanded T_reg_ cells. In vitro, we show that *IL-32* was significantly downregulated in the presence of the BACH2 inhibitor (*P* = 0.0318, *t* = 2.596, d.f. = 8; two-tailed independent *t*-test), but expression was recovered after IL-2 treatment (Fig. 6e). Similarly, IL-2 treatment maintained the gene expression of canonical T_reg_ cell functional genes including *IL-10* (Fig. 6e), *FOXP3* and *TGFB1* in the presence of the BACH2 inhibitor (Extended Data Fig. 9). In summary, IL-2 treatment maintains and promotes the human T_reg_ cell program, bypassing the potential detrimental effects of BACH2 suppression on T_reg_ cell homeostasis.

## Discussion

The utility of IL-2_LD_ as a T_reg_ cell-promoting immunomodulatory therapy is being investigated in a range of immune-mediated inflammatory diseases^41^. Despite this, there is still limited understanding of the specific impacts of this potential immunotherapy on T_reg_ cells in these patients. Our study analyzed scRNA-seq data from the LILACS trial to provide deeper insights into the biology involved in IL-2-dependent regulation of the T_reg_ cell response, in turn providing critical data into the mechanisms by which IL-2_LD_-based, T_reg_ cell-promoting therapies may benefit patients.

The scRNA-seq data showed that IL-2_LD_ caused the expansion of T_reg_ cells while contracting the naive CD4^+^ T cell compartment and having a minimal effect on CD4^+^ T_eff_ cells. Although the expanded T_reg_ cells still make up a relatively small proportion of T cells, their impact on inflammatory pathology is known to be oversized^42^. In the post-acute MI group treated with placebo, there was a limited clonal expansion of CD4^+^ T cells between the two timepoints, while IL-2_LD_ treatment markedly changed the TCR landscape. We showed that IL-2_LD_ caused T_reg_ cells to undergo clonal expansion, leading to decreased repertoire diversity. The 1.5 MIU d^−1^ dose caused the largest decrease in clonal diversity probably owing to its selectivity for T_reg_ cells, whereas the 2.5 MIU d^−1^ dose appears to have broader effects on T cells, potentially driving polyclonal expansion. Interestingly, although the 2.5 MIU d^−1^ dose had no significant effect on CD4^+^ T_em_ cell numbers in blood as assessed via flow cytometry and scRNA-seq, there is evidence that it caused more T_em_ cell clonal expansion than the 1.5 MIU d^−1^ dose. These data further support, although a posteriori, the selection of the 1.5 MIU d^−1^ dose for the phase 2b Low-Dose Interleukin 2 for the Reduction of Vascular Inflammation in Acute Coronary Syndromes (IVORY) trial^43^.

Our findings are supported by the paired scRNA-seq and scTCR-seq analyses with both TCR tracking and the more conventional trajectory analysis showing a T cell compartment-wide polarization toward a regulatory, immunosuppressive phenotype after IL-2_LD_ treatment. Specifically, it appears that IL-2_LD_ rescues the post-MI inflammatory T cell skew observed in the placebo group. This concept of T cell phenotyping switching in the post-MI context is supported by preclinical data^44,45^. This TCR tracking approach, using the adaptive immune receptor to identify related cells over time (and their linked phenotype), has implications beyond cardiovascular disease, offering more granular, clinically relevant conclusions than pseudotime-based techniques alone. We emphasize that this approach is not tracking individual T cells across timepoints but instead identifies changes in T cell phenotypes among highly related cells (that is, having the same TCR clonotype) over the two timepoints.

The antigen specificity prediction further confirms the clonal expansion of IL-2_LD_-treated T_reg_ cells, as the large cluster of expanded T_reg_ cells resolved by GLIPH2 identifies a sizable population of expanded T_reg_ cells with highly related TCRs, a finding subsequently corroborated via CoNGA analysis. The emergence of such a large T_reg_ cell cluster was unexpected given that T_cm_ and T_em_ cells comprise a larger proportion of the T cell compartment in the peripheral blood^46^. Furthermore, we show that the clonally expanded T_reg_ CDR3s cluster with motifs from proven atherosclerosis-related TCRs. Importantly, a much lesser degree of clustering is seen between the expanded T_reg_ cells and TCR motifs from healthy controls. Accordingly, we provide evidence that IL-2_LD_-induced clonal expansion involves specific motifs that are associated with atherosclerosis pathogenesis. However, we acknowledge that reactivity toward specific antigens will need to be verified experimentally in future studies.

By validating the induced regulatory phenotype of IL-2_LD_-expanded T_reg_ cells via ligand–receptor analysis, we confirmed maintenance of canonical T_reg_ cell immunosuppression after IL-2_LD_ treatment and highlighted potential in vivo mechanisms of action. The upregulation of TGF-β signaling pathways alongside well-characterized immune checkpoint receptor interactions such as LAG3 and PD-1 in patients with expanded T_reg_ cells points to a critical role for these cells in modulating the inflammatory milieu after MI.

The immunosuppressive potential of IL-2_LD_-expanded T_reg_ cells was associated with upregulation of genes involved in migration and homing to inflammatory tissues. In particular, the selective upregulation of CXCR3 suggests increased potential of the expanded T_reg_ cells to infiltrate tissues characterized by high expression of T_H_1-dependent and gamma-interferon-inducible CXCR3 ligands, including CXCL9, CXCL10 and CXCL11. This is precisely the case in atherosclerotic plaques^47–49^ and inflammatory cardiac tissue^50^, which are characterized by a T_H_1-mediated immune response, making IL-2_LD_ highly suitable for inflammation resolution in patients with atherosclerotic and ischemic heart disease. The immunosuppressive and migratory phenotype of IL-2_LD_-expanded T_reg_ cells of patients with ACS is consistent with the gut- and skin-homing T_reg_ cell phenotype described recently in patients with systemic lupus erythematosus after treatment with IL-2_LD_ (ref. ^11^), suggesting a direct role of IL-2_LD_ in generating immunosuppressive T_reg_ cells with increased homing potential to inflammatory tissues. In mice, glycolysis has been involved in the generation of migratory T_reg_ cells^51^, which is consistent with the upregulation of *ENO1* and *GAPDH* in our IL-2_LD_-expanded T_reg_ cells. Interestingly, the IL-2_LD_-expanded T_reg_ cells also maintain oxidative phosphorylation, which could be related to increased *FOXP3* (refs. ^52,53^) and *TGFB1* expression^54^. This may allow the tissue-infiltrating T_reg_ cells to maintain their suppressive potential in a low-glucose, high-lactate inflammatory environment. IL-2_LD_-expanded T_reg_ cells were enriched in effector T_reg_ cells, which is also consistent with the immunosuppressive and tissue-homing characteristics of these cells, as shown with the effector phenotype of T_reg_ cells that infiltrate arthritic inflammatory joints^55^ and atherosclerotic lesions in humans^56^.

The transcription factor BACH2 is understood to be an important regulator of T_reg_ cell differentiation and effector programs. Specifically, BACH2 has been identified as a repressor of T_eff_ cell programs^37^ and a suppressor of IL-2 signaling^40^. However, in the context of chronic disease such as cancer, BACH2 has been shown to drive T_reg_ cell quiescence and maintenance of immune homeostasis^39^. In cardiovascular disease and other immuno-inflammatory diseases, the role of BACH2 in regulating immune homeostasis is incompletely understood. Using SCENIC, we identify a network of regulators including *SATB1*, *LEF1* and *BACH2* that are differentially expressed (downregulated) in IL-2_LD_-expanded T_reg_ cells. This finding is very consistent with the decreased expression of *LEF1* in T_reg_ cells of human arthritic joints and human atherosclerotic plaques compared with PBMC-derived T_reg_ cells^55,56^, and with the role of *SATB1*, *LEF1* and *BACH2* in regulating the effector T_reg_ cell phenotype^57–61^. However, it is important to note that while the downregulation of these regulators promotes the effector T_reg_ cell phenotype, the same regulators are required for T_reg_ cell survival and maintenance^39,60,61^. Therefore, their sustained downregulation in T_reg_ cells may be harmful in the long term, as suggested by the autoimmune phenotype of mice with deletion of LEF1 and BACH2 in T_reg_ cells^40,61^. In this context, our finding that BACH2 may act as a regulator of the IL-2_LD_-dependent T_reg_ cell expansion program led us to examine the interaction of IL-2 with BACH2 inhibition in human T_reg_ cells. We found that BACH2 inhibition attenuated the *IL-32* expression characteristic of the expanded T_reg_ cells, but IL-2 treatment bypassed this effect to restore expression levels. A similar trend was observed for canonical T_reg_ cell genes under the same conditions, in which IL-2 treatment combined with BACH2 inhibition either restored or led to higher average transcript expression levels. The corroborating pSTAT5 data further suggest that BACH2 plays a multifaceted role in attenuating STAT5 phosphorylation and maintaining T_reg_ cell suppression, but that IL-2 treatment overrides these inhibitory effects in the T_reg_ cell expansion program. These findings may have important therapeutic implications. It has previously been shown that T_reg_ cell numbers and functions in diseased cardiovascular tissues become altered with disease progression, as is the case in human atherosclerotic plaques^62^ and ischemic cardiomyopathy^45^. We suggest that this is due, at least in part, to the inability of effector T_reg_ cells to survive in the long term and maintain immunosuppression owing to their reduced expression of LEF1 and BACH2. Our finding that IL-2 bypasses BACH2 downregulation to maintain the T_reg_ cell program strongly supports the clinical relevance of the IL-2_LD_ therapeutic approach, which will be needed to maintain tissue-resident effector T_reg_ cells and prevent their exhaustion. Previously, we have shown that BACH2 downregulation in B cells of patients treated with IL-2_LD_ promoted a B_reg_ cell phenotype^63^, further highlighting the role of this transcription factor in IL-2_LD_-induced immunomodulation.

There are several limitations of this study. Firstly, this work is based on scRNA-seq and scTCR-seq and therefore fallible owing to the shortcomings of this technology, which has been previously described^64^. Successful reconstitution of productive αβ chains on T cells in our experiments is 60%. This is similar to other datasets but does introduce the possibility that there is bias in the type of T cells that we further analyzed. We studied 16 patients, 32 samples and over 197,000 individual immune cells. Through the lens of single-cell transcriptomics, this represents a large dataset; however, in terms of the impact on human atherosclerosis biology, these findings will need to be confirmed clinically in a wider range of patients. The samples we have analyzed are taken from peripheral blood and may not fully reflect the immune dynamics seen in the disease tissue microenvironment.

In conclusion, our results lend important mechanistic insight and the molecular signatures of IL-2-expanded T_reg_ cells in the context of acute MI. These IL-2-responsive, immunosuppressive and tissue-migratory T_reg_ cells represent an important therapeutic target for CVD and related chronic inflammatory diseases.

## Methods

### LILACS trial

The methods from the LILACS trial (NCT03113773) have been previously published^10^. In summary, LILACS was a dual-center, randomized, double-blind, placebo-controlled clinical trial of low-dose IL-2 in patients with ACS approved by the UK Greater Manchester Central Research Ethics Committee and the UK Medicines and Healthcare Products Regulatory Agency (UK REC: 17/NW/0012). In Part B, there were 3 groups: saline (4 patients), 1.5 MIU d^−1^ IL-2 (6 patients) and 2.5 MIU d^−1^ (6 patients). PBMCs were collected before and after 5 days of treatment and stored in liquid nitrogen.

### Single-cell RNA sequencing

Detailed methodology for the 5′ 10X sequencing experiment has been published^10^. Briefly, PBMCs were thawed and enriched for live cells. Samples were loaded onto a 10x chip with the intention of recovering approximately 10,000 cells per lane. The library preparation was performed in accordance with manufacturer instructions and the libraries sequenced using Illumina chemistry at 2 × 150 bp. In this study, the T cells identified in the previous analysis were separated along with the immune receptor data. Immune receptor data were analyzed with scirpy, and only cells with single paired TCRs were brought forward for analysis. The primary analysis for the scRNA-seq data was performed using anndata v0.9.1, pandas v2.2.2, scanpy v1.9.3, scirpy v0.16.1, pysankey v1.4.2, pySCENIC v0.12.1, multinichenetr v1.0.0 and cellrank v2.0.0.

### Clonotype definition

Invariant TCRs were removed from the analysis in addition to the removal of cells with multiple TCR chains or orphan chains. Clonotype IDs were then assigned to the remaining TCRs using scirpy’s ‘define_clonotype_clusters’ function. The amino acid sequence was used, all receptor arms were considered and an aligned metric parameter was used (that is, distance was based on pairwise sequence alignments using the BLOSUM62 matrix) with a cut-off value of 10. V gene matching was not enforced in the definition.

### T_reg_ cell phenotyping, clonotype matching and TCR tracking

The clonotype size for the subset of T_reg_ cells post-IL-2_LD_ treatment was calculated. Briefly, the number of T_reg_ cells with a matching clonotype ID was counted, and a T_reg_ cell clonotype was defined as expanded if the clonotype size was ≥2.

Differential gene expression was then performed using scanpy (using the Wilcoxon ranked sum method). Using the differentially expressed genes from the expanded T_reg_ cells, a pathway enrichment analysis was performed; this was achieved using GSEApy’s enrichR function. A GSEA was performed on the log normalized gene expression data from the expanded and non-expanded T_reg_ cell subsets using the GSEApy signal-to-noise method. Both GSEA and enrichment analysis used the following predefined gene sets: Gene Ontology (GO):Biological Process, GO:Molecular Function, Molecular Signatures Database (MSigDB):Hallmark and Kyoto Encyclopedia of Genes and Genomes (KEGG) (https://maayanlab.cloud/enrichr/).

### Cell–cell interaction analysis

The MultiNicheNet analysis evaluated posttreatment timepoint samples from patients who received IL-2_LD_ treatment. The patients were divided into those who had expanded T_reg_ cell clones and those who did not as defined previously. A contrast matrix between the two groups was generated and the analysis performed using R version 4.2.1.

### T_reg_ cell culture

Primary human T_reg_ cells were isolated from fresh (collected within 12 h) apheresis cones using a Lymphoprep gradient and a subsequent EasySep Human CD4^+^CD127^low^CD25^+^ Regulatory T Cell Isolation Kit (Stemcell) or Human CD4^+^CD25^+^CD127^dim/−^ Regulatory T Cell Isolation Kit II (Miltenyi). Cells were cultured in RMPI media supplemented with 10% heat-inactivated FBS and 1% penicillin–streptomycin.

For the RT-qPCR experiments, cells were cultured for 24 h at 37 °C with IL-2-treated conditions being supplemented with 50 IU recombinant human IL-2 (R&D Systems). A BACH2 inhibitor (RGFP966, Selleckchem) was added at 10 μM in the BACH2-inhibition conditions. After 24 h, cells were transferred to a v-bottom plate, the supernatant frozen at −80 °C and the cells lysed using RLT Plus lysis buffer (Qiagen). RNA was then extracted using the RNEasy Micro Plus kit (Qiagen) and the concentration measured using a Qubit HS RNA kit (Qiagen). RNA amounts were normalized based on the Qubit values, then reverse transcription was performed using a Quantitect kit (Qiagen).

For the pSTAT5, CD122 or CD25 experiments, T_reg_ cells were stimulated with anti-CD3 and anti-CD28 beads. After 48 h of initial stimulation, the cells were collected, washed three times in RPMI medium and then cultured in RPMI medium without serum, with or without the BACH2 inhibitor, for 8 h. The IL-2-treated cells were subsequently stimulated with recombinant human IL-2 at 50 IU ml^−1^ for 15 min.

### RT-qPCR

From the cDNA generated in the reverse transcriptase reaction, RT-qPCR was performed using the following TaqMan probes: TGFB1 (Hs00998133_m1), BACH2 (Hs00935338_m1), IL-32 (Hs00992441_m1), FOXP3 (Hs01085834_m1), IL-10 (Hs00961622_m1) and Eukaryotic 18S rRNA Endogenous Control (all Thermo Fisher). RT-qPCRs were performed using TaqMan Fast Advanced Master Mix (Thermo Fisher) and Lightcycler 480 II (software v1.5.0) (Roche). All reactions were performed at a final cDNA dilution of 1:10.

### Flow cytometry for pSTAT5

Pelleted cells were suspended in 500 µl of ice-cold PBS. A total of 500 µl of 4% PFA (final concentration is 2% PFA) was added to each tube, and the contents were mixed gently and vortexed. The tubes were incubated on ice for 30 min. Then, 2 ml of ice-cold PBS was added to each tube, and then the tubes were centrifuged at 4 °C. The supernatant was discarded, and 2 ml of ice-cold PBS was added to each tube, followed by centrifugation. The supernatant was discarded and 1 ml of prefrozen (−80 °C) 100% methanol was added to each tube. Tubes were incubated in a freezer at −80 °C for 30 min. Then, 2 ml of ice-cold 10% FCS–PBS was added to the cells, and the cells were spun down at 4 °C. The supernatant was discarded and the cells washed again. Subsequently, 2 ml of cold FACS buffer was added to each tube, and the cells were spun down and resuspended in 200 µl of FACS buffer and 1 µl of pSTAT5 antibody. The cells were gently mixed and incubated in FACS tubes at 4 °C for 30 min. Analysis was done using FACSDiva software v9.0 (BD) and displayed dot plots and histograms were obtained using FlowJo v10.10 software (FlowJo, LLC). The relevant gating scheme is included in Supplementary Fig. 1. The following manufacturer-conjugated antibodies were used at a dilution of 1:200: BV650 CD3 (clone: OKT3, catalog number: 317324, lot: B259475; BioLegend), FITC CD4 (clone: RPA-T4, catalog number: 300506, lot: B264353; BioLegend), BV421 CD25 (clone: S20019D, catalog number: 385208, lot: B404986; BioLegend), BV605 CD127 (clone: A019D5, catalog number: 351334, lot: B256273; BioLegend) and PE-pSTAT5 (clone: Al7016B.Rec, catalog number: 936904, lot: B380798; BioLegend). Each antibody was validated with a series of titration dilutions, certification of which can be accessed by lot number at https://www.biolegend.com.

### Statistics and reproducibility

The scRNA-seq analyses of the LILACS trial were prespecified exploratory studies. Here we present the scRNA-seq from 16 patients from Part B of the trial. Patients were randomly assigned to treatment with either placebo or aldesleukin. The trial was double blinded.

Unless otherwise stated, analyses used *α* = 0.05 as a cut-off for statistical significance, adjusting for multiple testing where relevant. Bioinformatic analyses used the standard parameters recommended by each individual package for statistical testing, unless otherwise stated. All in vitro experiments used a minimum of three biological replicates to facilitate statistical analysis and ensure reproducibility.

### Reporting summary

Further information on research design is available in the Nature Portfolio Reporting Summary linked to this article.

## Supplementary information

Supplementary Information Supplementary Fig. 1 and discussion of Extended Data Fig. 5.

Reporting Summary

Supplementary Tables Supplementary Tables 1–5.

## Source data

Source Data Fig. 6 Source data for Fig. 6d,e.

Source Data Extended Data Fig. 9 Source data for Extended Data Fig. 9.

## Data Availability

The data from the LILACS trial presented in this study will be shared on reasonable request and in compliance with the UK General Data Protection Regulation (GDPR) owing to the data confidentiality of living subjects and ethical and/or legal issues. Requesters will be required to sign a data access agreement to ensure the appropriate use of the study data. Requests can be sent to corresponding author T.X.Z.

## Code availability

All code used to perform the described analysis is available via GitHub at https://github.com/zhaolabcambridge/Case_et_al_2025_NCVR.
