# Supplementary material for: Low-dose interleukin-2 induces clonal expansion of BACH2-repressed effector regulatory T cells following acute coronary syndrome
Source: Nat Cardiovasc Res. 2025 Jun 3;4(6):727–39. doi: 10.1038/s44161-025-00652-y (PMC12170346; doi:10.1038/s44161-025-00652-y)
Supplement: Supplementary file 1 — Supplementary Fig. 1 and discussion of Extended Data Fig. 5. [file 44161_2025_652_MOESM1_ESM.pdf]

nature cardiovascular research

Supplementary information

https://doi.org/10.1038/s44161-025-00652-y

Low-dose interleukin-2 induces clonal expansion of BACH2-repressed effector regulatory T cells following acute coronary syndrome

In the format provided by the authors and unedited

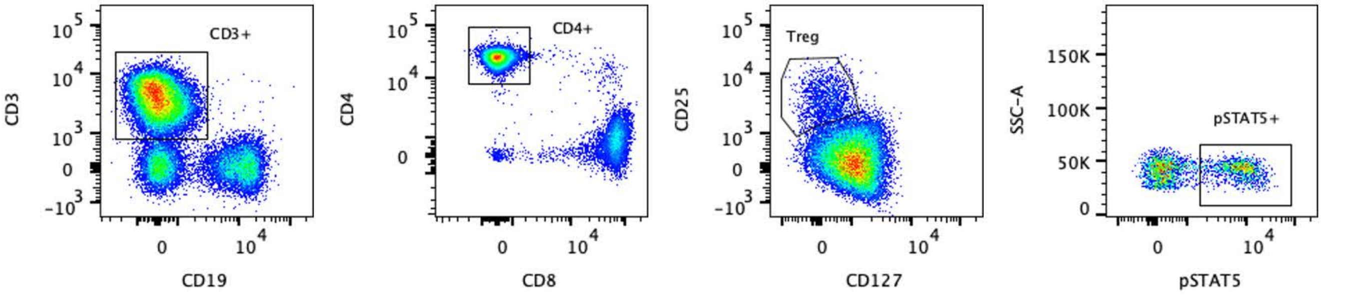

Supplementary Figure 1: Detailed gating strategy for data in Figure 6C and 6D.

Supplementary Explanation and Interpretation of Extended Data Figure 5.

Using the UMAP gene overlays, we can see that clusters 0, 2, 10, and 14 contain $CD4^{+}$ Tregs, as they express relatively high levels of *FOXP3* and *IL2RA*. We see that two significant hits are from GEX clusters 2 and 0 (which we map as Tregs based on the above rationale), and both map to a single TCR cluster: 0. We show that the cells in this cluster are predominately IL-2_{LD} treated and are enriched in clonally expanded Tregs (compared to other GEX clusters containing Tregs). The enriched genes confirm that the cells in this CoNGA cluster are associated with the expanded Treg phenotype described elsewhere in this study, with differential expression of genes including *LGALS3* and *IL32*.
