# Supplementary material for: Low-dose interleukin-2 induces clonal expansion of BACH2-repressed effector regulatory T cells following acute coronary syndrome
Source: Nat Cardiovasc Res. 2025 Jun 3;4(6):727–39. doi: 10.1038/s44161-025-00652-y (PMC12170346; doi:10.1038/s44161-025-00652-y)
Supplement: Supplementary file 2 — Reporting Summary [file 44161_2025_652_MOESM2_ESM.pdf]

nature portfolio

Corresponding author(s): Tian X Zhao, Ziad Mallat

Last updated by author(s): Mar 24, 2025

Reporting Summary

Nature Portfolio wishes to improve the reproducibility of the work that we publish. This form provides structure for consistency and transparency in reporting. For further information on Nature Portfolio policies, see our Editorial Policies and the Editorial Policy Checklist.

Statistics

For all statistical analyses, confirm that the following items are present in the figure legend, table legend, main text, or Methods section.

n/a	Confirmed	
☐	☒	The exact sample size (*n*) for each experimental group/condition, given as a discrete number and unit of measurement
☐	☒	A statement on whether measurements were taken from distinct samples or whether the same sample was measured repeatedly
☐	☒	The statistical test(s) used AND whether they are one- or two-sided<br>*Only common tests should be described solely by name; describe more complex techniques in the Methods section.*
☒	☐	A description of all covariates tested
☐	☒	A description of any assumptions or corrections, such as tests of normality and adjustment for multiple comparisons
☐	☒	A full description of the statistical parameters including central tendency (e.g. means) or other basic estimates (e.g. regression coefficient) AND variation (e.g. standard deviation) or associated estimates of uncertainty (e.g. confidence intervals)
☐	☒	For null hypothesis testing, the test statistic (e.g. *F*, *t*, *r*) with confidence intervals, effect sizes, degrees of freedom and *P* value noted<br>*Give P values as exact values whenever suitable.*
☒	☐	For Bayesian analysis, information on the choice of priors and Markov chain Monte Carlo settings
☒	☐	For hierarchical and complex designs, identification of the appropriate level for tests and full reporting of outcomes
☒	☐	Estimates of effect sizes (e.g. Cohen's *d*, Pearson's *r*), indicating how they were calculated

Our web collection on statistics for biologists contains articles on many of the points above.

Software and code

Policy information about availability of computer code

Data collection	BD FACSDiva (v9.0) and Roche LightCycler 480 (v1.5.0) were used.
Data analysis	The primary analysis for the scRNAseq data was performed using anndata v0.9.1, pandas v2.2.2, scanpy v1.9.3, scirpy v0.16.1, pysankey v1.4.2, pySCENIC v0.12.1, multinichenetr v1.0.0, and cellrank v2.0.0.

For manuscripts utilizing custom algorithms or software that are central to the research but not yet described in published literature, software must be made available to editors and reviewers. We strongly encourage code deposition in a community repository (e.g. GitHub). See the Nature Portfolio guidelines for submitting code & software for further information.

Data

Policy information about availability of data

All manuscripts must include a data availability statement. This statement should provide the following information, where applicable:

- Accession codes, unique identifiers, or web links for publicly available datasets
- A description of any restrictions on data availability
- For clinical datasets or third party data, please ensure that the statement adheres to our policy

The data from the LILACS trial presented in this study will be shared in group form, on reasonable request and in compliance with the UK General Data Protection Regulation (GDPR), due to data confidentiality of living subjects and ethical and/or legal issues. Requests for data should be directed to TXZ (txz20@cam.ac.uk). Requesters will be required to sign a data access agreement to ensure the appropriate use of the study data.

Research involving human participants, their data, or biological material

Policy information about studies with human participants or human data. See also policy information about sex, gender (identity/presentation), and sexual orientation and race, ethnicity and racism.

Reporting on sex and gender	Findings from this work apply to both sexes. The study was designed to recruit patient male and female patients with acute myocardial infarction. Sex was determined by self reporting. Sex data is reported in aggregate in the original publication - Zhao et al. NEJM 2022. They are not re-described in this submitted publication. Sex based analysis was not performed as this was not a pre-specified analysis, furthermore numbers of patients are too low to make conclusions based on subgroups.
Reporting on race, ethnicity, or other socially relevant groupings	Ethnicity was determined by self-reporting. This data is reported in aggregate in the original publication (Zhao et al. NEJM Ev 2022). They are not re-described in this submitted publication.
Population characteristics	Population characteristics are reported in aggregate in the original publication (Zhao et al. NEJM Ev 2022). They are not re-described in this submitted publication.
Recruitment	Patient recruitment for the LILACS study has been published (Zhao et al. NEJM 2022) and not described in the current publication. Potential patients was identified by their treating clinicians / nurses or the trial team. Sources for these potential patients included outpatient clinic visits / lists , inpatients wards, and research databases. Initial contact was made by the clinician who knows the patient or is currently looking after the patient . The contact details of identified potential patients, with their agreement, were passed to the trial team to enable them to directly contact the potential patient.
Ethics oversight	The trial was approved by the UK Greater Manchester Central Research Ethics Committee and the UK Medicines and Healthcare Products Regulatory Agency.

Note that full information on the approval of the study protocol must also be provided in the manuscript.

Field-specific reporting

Please select the one below that is the best fit for your research. If you are not sure, read the appropriate sections before making your selection.

☒ Life sciences ☐ Behavioural & social sciences ☐ Ecological, evolutionary & environmental sciences

For a reference copy of the document with all sections, see nature.com/documents/nr-reporting-summary-flat.pdf

Life sciences study design

All studies must disclose on these points even when the disclosure is negative.

Sample size	This is an exploratory study that is not designed to formally test a hypothesis in a confirmatory fashion. Given that both parts of the trial have clinical safety as primary endpoints, a formal power calculation is not relevant. A sample size of 41 patients is achievable within the proposed time scale, given the size of the targeted patient population at our study site.
Data exclusions	None.
Replication	The main publication included 41 patients. Here we present the scRNA-seq from 16 patients from Part B of the trial.
Randomization	This has been published in the Protocol paper (Zhao et al. BMJ Open 2018) and the results manuscript (Zhao et al. NEJM Ev 2022). Patients were randomly assigned to either treatment with placebo or aldesleukin. The trial was double blinded. Randomisation was carried out via a paper-based concealment list generated by a statistician.
Blinding	To maintain the overall quality and legitimacy during the clinical trial, unblinding was only occur in exceptional circumstances when knowledge of the actual treatment is essential for further clinical management of the patient. This never occurred. Analysis of the results was done in blinded manner with treatment allocation known only as A and B. Unblinding occurred after analysis was completed.

Reporting for specific materials, systems and methods

We require information from authors about some types of materials, experimental systems and methods used in many studies. Here, indicate whether each material, system or method listed is relevant to your study. If you are not sure if a list item applies to your research, read the appropriate section before selecting a response.

Materials & experimental systems

n/a	Involved in the study	
☐	☒	Antibodies
☒	☐	Eukaryotic cell lines
☒	☐	Palaeontology and archaeology
☒	☐	Animals and other organisms
☐	☒	Clinical data
☒	☐	Dual use research of concern
☒	☐	Plants

Methods

n/a	Involved in the study	
☒	☐	ChIP-seq
☐	☒	Flow cytometry
☒	☐	MRI-based neuroimaging

Antibodies

Antibodies used	BV650 CD3, Clone: OKT3,Cat No: 317324, Lot: B259475, BioLegend<br>FITC CD4, Clone: RPA-T4, Cat No: 300506, Lot: B264353, BioLegend<br>BV421 CD25, Clone: S20019D, Cat No: 385208, Lot: B404986, BioLegend<br>BV605 CD127, Clone: A019D5, Cat No: 351334, Lot: B256273, BioLegend<br>PE pSTAT5, Clone: A17016B.Rec, Cat No: 936904, Lot: B380798, BioLegend<br>All antibodies used at 1:200 dilution.
Validation	Each antibody was validated with a series of titration dilutions, certification of which can be accessed by lot number on biolegend.com.

Clinical data

Policy information about clinical studies

All manuscripts should comply with the ICMJE guidelines for publication of clinical research and a completed CONSORT checklist must be included with all submissions.

Clinical trial registration	The trial was registered on Clinicaltrials.gov (NCT03113773) prior to trial commencement
Study protocol	Published: Zhao et al. BMJ Open 2018
Data collection	Between May 2017 and February 2019, patients at Cambridge University Hospital and Royal Papworth Hospital were recruited for this study
Outcomes	For part A, the primary outcome was safety; in part B, the coprimary endpoints were safety and estimating the dose of aldesleukin to increase Tregs by 75% frombase line to follow-up (V7). For both study phases, safety was assessed by an open query for adverse events (AEs), a physical examination, a review of concomitant medications, vital signs, and safety blood tests performed at all visits. Additionally, electrocardiograms (predose and 15, 30, and 60 minutes postdose) and cardiac telemetry were performed at dosing visits, and echocardiography was performed at screening and on final follow-up. In part B, Tregs were defined as CD3+CD4 +CD25highCD127low and expressed as the percentage of total CD4+ T cells in peripheral blood. Additional information on prespecified exploratory end points, including changes in lymphocyte subsets, cardiac and inflammatory biomarkers, and scRNA-seq of PBMCs can be found in the results manuscript (Zhao et al NEJM Ev 2022)

Plants

Seed stocks	No plants involved; form error.
Novel plant genotypes	No plants involved; form error.
Authentication	No plants involved; form error.

Flow Cytometry

Plots

Confirm that:

☒ The axis labels state the marker and fluorochrome used (e.g. CD4-FITC).

☒ The axis scales are clearly visible. Include numbers along axes only for bottom left plot of group (a 'group' is an analysis of identical markers).

☒ All plots are contour plots with outliers or pseudocolor plots.

☒ A numerical value for number of cells or percentage (with statistics) is provided.

Methodology

Sample preparation	Pelleted primary human Tregs were suspended in 500 µL ice-cold PBS. 500 µL 4% PFA (final concentration is 2% PFA) was added to each tube, mixed gently, and vortexed. The FACS tubes were incubated on ice for 30 min. 2 mL of ice-cold PBS was added to each tube, and then the tubes were centrifuged at 4ºC. The supernatant was discarded, and 2 mL of ice-cold PBS was added to each tube, followed by centrifugation. The supernatant was discarded and 1 mL of pre-frozen (-80ºC) 100% methanol was added to each tube. Tubes were incubated in the -80ºC freezer for 30 min. 2 mL of ice-cold 10% FCS-PBS was added to cells, and cells were spun down at 4ºC. The supernatant was discarded and the cells washed again. 2mL of cold FACS buffer was added to each tube, then cells were spun down and resuspended in 200 µL of FACS buffer and 1 µL of pSTAT5 antibody. Cells were gently mixed and incubated in FACS tubes at 4ºC for 30 min.
Instrument	BD FACSymphony A3
Software	BD FACSDiva (v9.0) was used for collection and FlowJo (v10.10) was used for analysis.
Cell population abundance	No FACS performed.
Gating strategy	Tregs were defined as CD3+ CD4+ CD25+ CD127 low cells, with all downstream analysis using this population.

☒ Tick this box to confirm that a figure exemplifying the gating strategy is provided in the Supplementary Information.
